# Gut-Liver-Brain Axis and Alcohol Use Disorder: Treatment Potential of Fecal Microbiota Transplantation

**DOI:** 10.35946/arcr.v44.1.01

**Published:** 2024-02-01

**Authors:** Jennifer T. Wolstenholme, Nikki K. Duong, Emily R. Brocato, Jasmohan S. Bajaj

**Affiliations:** 1 Alcohol Research Center, Virginia Commonwealth University, Richmond, Virginia; 2 Department of Pharmacology and Toxicology, Virginia Commonwealth University, Richmond, Virginia; 3 Division of Gastroenterology, Hepatology and Nutrition, Virginia Commonwealth University, Richmond, Virginia; 4 Central Virginia Veterans Healthcare System, Richmond, Virginia

**Keywords:** alcohol, fecal microbiota transplant, alcohol-associated liver disease, gut-brain axis, gastrointestinal microbiome, microbiota, probiotics, behavior

## Abstract

**PURPOSE:**

Chronic alcohol use is a major cause of liver damage and death. In the United States, multiple factors have led to low utilization of pharmacotherapy for alcohol use disorder (AUD), including lack of provider knowledge and comfort in prescribing medications for AUD. Alcohol consumption has direct effects on the gut microbiota, altering the diversity of bacteria and leading to bacterial overgrowth. Growing evidence suggests that alcohol’s effects on the gut microbiome may contribute to increased alcohol consumption and progression of alcohol-associated liver disease (ALD). This article reviews human and preclinical studies investigating the role of fecal microbiota transplantation (FMT) in ameliorating alcohol-associated alterations to the liver, gut, and brain resulting in altered behavior; it also discusses the therapeutic potential of FMT.

**SEARCH METHODS:**

For this narrative review, a literature search was conducted in September 2022 of PubMed, Web of Science Core Collection, and Google Scholar to identify studies published between January 2012 and September 2022. Search terms used included “fecal microbiota transplantation” and “alcohol.”

**SEARCH RESULTS:**

Most results of the literature search were review articles or articles on nonalcoholic fatty liver disease; these were excluded. Of the remaining empirical manuscripts, very few described clinical or preclinical studies that were directly investigating the effects of FMT on alcohol drinking or related behaviors. Ultimately, 16 studies were included in the review.

**DISCUSSION AND CONCLUSIONS:**

The literature search identified only a few studies that were directly investigating the effect of FMT on ALD or alcohol drinking and related behaviors. Largely proof-of-concept studies, these findings demonstrate that alcohol can alter the gut microbiome and that the microbiome can be transferred between humans and rodents to alter affective behaviors frequently associated with increased alcohol use. Other studies have shown promise of FMT or other probiotic supplementation in alleviating some of the symptoms associated with ALD and drinking. These results show that the implementation of FMT as a therapeutic approach is still in the investigatory stages.

Alcohol-associated liver disease (ALD) is a leading cause of morbidity and mortality in people with alcohol use disorder (AUD).^1^ Alcohol exerts its effect on the liver through both direct and indirect pathways and can eventually lead to steatosis, steatohepatitis, fibrosis, hepatocellular carcinoma, and cirrhosis.^2^ However, only approximately 10% to 20% of patients with ALD develop cirrhosis.^2^ When decompensated cirrhosis develops, liver transplantation should be considered; however, a transplant may not be a feasible option for certain patients. Transplant eligibility is determined in a multidisciplinary fashion that includes a vigorous medical, psychosocial, surgical, and financial evaluation. Furthermore, the peri- and post-transplant periods can pose unique challenges to patients with underlying AUD. Individuals with chronic AUD are at risk for nutrient deficiencies, malnourishment, and sarcopenia.^3^ As such, they can enter transplant in a frail state that can predispose patients to infection, impaired wound healing, and sarcopenia (loss of muscle mass and function). In addition, transplant committees often require that patients engage in post-transplant alcohol cessation programs. To obviate the need for liver transplants, efforts to treat AUD and reduce craving should begin earlier in the disease course. In the United States, currently approved pharmacologic therapies for AUD include disulfiram, acamprosate, and naltrexone.^4^

Although pharmacological treatments exist, the treatment gap for AUD is higher than for any other mental disorder,^5^ and these treatments are prescribed only for a small percentage of patients with AUD. Several factors may contribute to the underuse of pharmacologic treatments for AUD, including lack of provider knowledge and comfort in prescribing these medications, low compliance with treatment among patients, and patient heterogeneity combined with the availability of only three approved medications. Thus, most patients with AUD—especially those with advanced AUD—are left untreated, and there is a need for additional, more effective therapies.

Newer therapeutic regimens include gut microbiome manipulation, which may modulate alcohol intake and drinking behavior.^2^,^6^ Growing evidence suggests that alteration of intestinal microbiota—which include not only bacteria but also fungi and viruses—contributes to the progression of excessive alcohol consumption and ALD, and this may form a therapeutic target.^2^,^6^ Alcohol consumption has both direct and indirect effects on the gut microbiota via alcohol metabolism, activation of inflammatory cascades, and alterations in the enteric nervous system.^2^,^6^ This suggests that by altering the gut microbiota, alcohol consumption may be modulated, slowing the progression of ALD.^2^,^6^

## The Impact of Alcohol on the Gut-Liver Axis

Gut-liver communication occurs both through the hepatic portal vein and the hepatic biliary system and can be influenced by the gut microbiota.^6^ Dietary nutrients absorbed from the gut can be carried directly to the liver via the portal vein. However, if the gut microbiota composition or gut barrier function is disrupted, other mediators or toxins can take the same route to disrupt liver homeostasis.^7^ The hepatic biliary system along with systemic circulation allows the liver to provide feedback to the gut via release of bile acids and other bioactive molecules.^6^

Alcohol consumption induces gut dysbiosis, an imbalance in gut microbiota, through several mechanisms. Chronic alcohol exposure decreases the production of mucus and antimicrobial peptides such as alpha-defensins and disrupts the intestinal barrier.^2^,^8^,^9^ This allows for translocation of lipopolysaccharide (LPS) and other endotoxins into the liver via the portal vein.^10^ LPS is produced by gram-negative bacteria and is one of the main factors in the pathogenesis of ALD. LPS activates toll-like receptors on the surface of Kupffer cells and induces pro-inflammatory signaling cascades, the release of cytokines, and, ultimately, hepatocyte damage.^6^ People with ALD often show higher levels of circulating pro-inflammatory mediators, such as LPS, interleukin 8 (IL-8), and IL-17.^11^ Pro-inflammatory circulating cytokines were found to positively correlate with scores of depression, anxiety, and alcohol craving in active drinkers.^12^ Moreover, inflammation markers were found to correlate with ALD severity.^7^,^13^

Alcohol use could also alter gut microbiota by reducing production of short-chain fatty acids (SCFAs), which are beneficial fermentation products.^14^ SCFAs have anti-inflammatory and immune-modulatory activity and help maintain the intestinal barrier.^6^ Alcohol has been shown to decrease SCFA production, reflected in the fecal content of patients with alcohol-associated cirrhosis.^15^ This alcohol-induced disruption of bacterial metabolites (such as SCFAs, and bile acids among others) is a consequence of altered gut microbiota composition.

Alcohol use has been shown to result in bacterial overgrowth and dysbiosis. In general, alcohol reduces *Bacteroidetes, Clostridia*, and *Verrucomicrobiae* and leads to increases in *Proteobacteria, Gammaproteobacteria*, and *Bacilli*.^16^ Alcohol also has direct cytotoxic effect on hepatocytes; its metabolite acetaldehyde triggers pro-inflammatory signaling cascades and damages the epithelial barrier.^9^

## The Impact of Alcohol on the Gut-Brain Axis

The gut microbiome also influences brain function and behavior through a variety of mechanisms and thus may be involved in the onset and severity of some psychiatric disorders, such as AUD.^6^ Research has suggested that bacterial metabolites can cross the blood-brain barrier via sensory nerves that innervate the gut.^6^ In patients with AUD, chronic low-grade inflammation leads to changes in pro-inflammatory mediators that can cross the blood-brain barrier to activate nuclear factor kappa B (NF-kB) in glial cells, leading to neuronal damage.^17^ This concept was further confirmed in a study demonstrating that a single injection of LPS led to increases in tumor necrosis factor-alpha (TNF-alpha) in the liver and brain, promoted microglial activation, and induced degeneration of dopamine-secreting neurons.^17^ Although some bacterial species can produce neurotransmitters, such as gamma-aminobutyric acid (GABA) and dopamine, it is debated whether these neurotransmitters can cross the blood-brain barrier.^6^ It may be that signaling by the vagal nerve influences neurotransmitter production, which could impact behaviors associated with AUD, such as anxiety.^6^ However, anti-inflammatory cytokines such as IL-10 have been shown to reverse anxiety-like behavior related to substance use.^18^ Thus, multiple factors can influence the development of mood disorders. Vagal signaling may play a critical role in the onset and severity of AUD, as significant reduction in voluntary drinking was seen in rats that underwent vagotomy.^19^

Microbiota-derived ammonia can also impact the central nervous system.^6^ Due to poor hepatic clearance, high levels of ammonia are seen in some patients with ALD, which can reach the brain and lead to astrocyte death, brain damage, and cognitive alterations. Another potential mechanism how gut microbiota may affect brain function is through the previously discussed alcohol-related decrease in levels of SCFAs, such as butyrate.^6^ Butyrate is a potent inhibitor of histone deacetylases and thus can lead to epigenetic changes such as modulation of histone modifications.^20^ Such epigenetic changes in the brain have the potential to impact current and future substance use by modulating addiction and reward networks.^21^ One study reported correlations between the gut microbiome and behavioral and neurophysiological traits that define AUD, such as measures of impulsivity and augmentations in striatal dopamine receptor expression.^22^

This review presents the growing number of clinical and preclinical studies that are beginning to investigate the therapeutic role and mechanisms underlying fecal microbiota transplantation (FMT) in ALD and AUD (see Table 1). It is important to note that not all patients with AUD have dysbiosis and/or increased intestinal permeability; the reason for this is unclear. A literature search using the terms “fecal microbiota transplantation” AND “alcohol” found very few studies that directly investigated the effect of FMT on alcohol drinking behavior. In addition, only a small number of articles showed the impact of FMT on affective behaviors that are frequently associated with excessive alcohol use. Some studies have shown promise in using gut microbial manipulation for alleviating some of the symptoms associated with ALD. Using these studies, the review outlines the interplay between the modulation of the gut microbiome, the gut-liver-brain axis, and AUD. The article also discusses why microbiome manipulation may be a promising therapeutic for ALD and proposes future directions.

## Search Methods

A September 2022 search of the PubMed database using the search terms “fecal microbiota transplantation AND alcohol, NOT review” identified 71 articles that were published between January 1996 and September 2022. Among these articles, 16 were preclinical studies that used alcohol in their model (e.g., animals treated with alcohol, or animals treated with FMT from alcohol-exposed subjects). Most of the excluded articles described studies of non–alcohol-associated liver disease. Of the 16 included preclinical publications, six assessed the effects of FMT or the modulation of the microbiome on ALD. Six other articles investigated the role of modulation of the gut microbiome on alcohol-associated behaviors (e.g., sociability, anxiety, and depression) or drinking behavior, with some reporting changes in gene or protein expression in the brains of recipient animals. The other four articles not directly discussed below were excluded for the following reasons: one article was a commentary, and three were focused on alcohol’s role on innate and adaptive immunity or pulmonary infection, not the gut-liver-brain axis. The 71 identified articles included 11 human/clinical studies, but four were excluded because they were either not related to alcohol or were not focused on microbial therapeutics. The remaining seven articles were human/clinical studies related to alcohol or cirrhosis (see Table 1).

A similar search strategy was employed in the Web of Science Core Collection database and Google Scholar. These searches identified 32 publications, and these were also contained in the PubMed dataset. Of note, none of these publications were published prior to 2016.

## Results

### Gut Microbiome and ALD: Preclinical Studies

In one of the seminal preclinical studies to investigate whether manipulation of the intestinal microbiome can prevent the development of ALD, Ferrere et al. showed that factors other than alcohol exposure are involved in the development of ALD.^23^ In this study that compared mice raised in two different institutions and that were fed the same Lieber-DeCarli diet—a liquid diet for rodents that contains all dietary and hydration needs as well as alcohol to induce the pathogenesis of early-stage ALD—mice consumed similar amounts of alcohol, had similar liver weights, and initially had similar fecal microbiota composition. However, mice from one facility developed early signs of ALD while mice from the other facility did not. Following 10 days of the Lieber-DeCarli diet supplemented with 5% ethanol, the animals exhibited specific microbiota profiles that were associated with susceptibility or resistance to ALD symptoms. In the ALD-sensitive mice, the alcohol diet induced a decrease of cecal *Bacteroidetes* and *Proteobacteria* and an increase of *Actinobacteria* and *Firmicutes*. Thus, the ALD-sensitive mice had 50% less *Bacteroides* than did the ALD-resistant mice at the end of the 10-day period. To prove that the microbiota were likely responsible for ALD sensitivity or resistance, the researchers performed FMT by transferring fecal matter from ALD-resistant mice to ALD-sensitive mice. FMT or pectin (complex heteropolysaccharides that can modulate the growth of gut microbiota) treatment protected the susceptible mice from alcohol-induced depletion of *Bacteroides*, and the microbiomes of FMT-treated mice were similar to the microbiomes of ALD-resistant mice. Moreover, FMT prevented the development of alcohol-induced liver lesions.^23^ This study was an important first step in showing that the endogenous microbiome influences an individual’s susceptibility to ALD and that manipulation of the intestinal microbiome can prevent the development of alcohol-induced liver lesions and may be a strong therapeutic treatment strategy.

Following this seminal study, additional research groups investigated whether probiotics or dietary supplements that alter the microbiome can also reduce ALD symptoms.^6^,^19^,^24^–^29^ These studies generally demonstrated a positive outcome of treatment with probiotics on liver outcomes; however, as they did not use FMT, a detailed discussion is beyond the scope of this article. To mechanistically understand how pectin alters the intestinal microbiome and therapeutically treats ALD, mice received an FMT from patients with severe alcohol-associated hepatitis to establish alcohol-induced liver lesions in the context of the human microbiota.^30^ The animals were then treated with pectin via FMT. Compared with control animals, pectin-treated mice showed a higher number of bacterial genes involved in carbohydrate, lipid, and amino-acid metabolism. Metabolomic analyses identified alterations in bacterial tryptophan metabolism and increased indole derivatives, suggesting activation of the aryl hydrocarbon receptor (AhR) signaling system. AhR agonists simulated the effects of pectin in liver tissue and reversed the signs of ALD. Conversely, knock-out of the AhR gene in mice reduced the effects of beneficial microbiota on alcohol-induced liver injury. Finally, the researchers found decreased level of AhR agonists in patients with severe alcohol-associated hepatitis, suggesting that AhR may be a new therapeutic target in ALD.^30^ These findings indicate that pectin reshapes the microbiome in the context of the human microbiota and not only prevents, but reverses, alcohol-induced liver injury in mice.

In another study, Yu et al. directly compared FMT to clustered interspaced short palindromic repeats (CRISPR) inactivation of low-density lipoprotein receptor-related protein 6 (LRP6), a co-receptor of the canonical Wnt/beta-catenin pathway, in their ability to ameliorate ALD symptoms.^31^ Knock-down of LRP6 by CRISPR, they hypothesized, would reduce Wnt signaling in hemopoietic stem cells to reduce their activation and, thus, improve the effects of liver fibrogenesis in their model of ALD. Rats fed an ethanol-containing Lieber-DeCarli diet to induce liver fibrosis and model early-stage ALD were then administered FMT from healthy rats or treated with LRP6-CRISPR. Histological and molecular assays revealed moderately improved liver histological markers in the FMT-treated rats that were accompanied by similar changes in fibrosis biomarkers. LRP6-CRISPR–treated mice showed similar improvements in liver histology and molecular markers, but with a greater effect size. Both LRP6-CRISPR and FMT treatment partially restored the composition of the gut microbiome and increased gut microflora diversity. Compared with untreated ALD-rats, LRP6-CRISPR and FMT both increased gut microbiota richness and diversity and resulted in a similar microbiota composition structure. Thus, principal coordinate analysis indicated that the gut microbiome of rats treated with LRP6-CRISPR and FMT overlapped and intersected with each other and with the control group. Specifically, LRP6-CRISPR and FMT each increased abundance of *Lactobacillus*. Thus, targeting the gut microbiome using samples from healthy rats or directly inactivating a member of the Wnt signaling pathway can improve the diversity and composition of the microbiome to ameliorate ALD symptoms.^31^

Three studies have used FMT procedures to show that gut microbiome remodeling may be a causal mechanism underlying the hepatoprotective effects and reductions in alcohol-induced liver injury of specific dietary enhancements, such as ursolic acid (UA) or Goji berries.^32^–^34^ UA, a bioactive constituent in teas, fruits, edible plants, and herbs, also has hepatoprotective activity.^35^–^36^ Using a model of chronic alcohol exposure to induce liver injury, Yan et al. showed that UA had not only hepatoprotective effects, but also suppressed alcohol-induced oxidative stress and intestinal barrier disruption.^33^ An FMT study was performed to investigate the possible contribution of gut microbiota manipulation in the beneficial effects of UA on alcohol-induced liver injury. Compared to mice receiving control-FMT, recipients of FMT from UA-consuming donors had a remodeled gut microbiome, less alcohol-induced gut dysbiosis, and reduced oxidative stress.^33^ Alcohol-induced liver injury was also partly alleviated in UA-FMT recipient mice, suggesting the hepatoprotective activity of UA is transferable and can be partly attributed to gut dysbiosis correction.^33^ Using a traditional Chinese medicinal plant, Goji berries, Guo et al. were able to restore the intestinal epithelial cell integrity and prevent acute liver injury induced by alcohol intake in mice.^34^ To examine whether the Goji-modulated gut microbiota played a causal role on liver protection, an FMT experiment was performed in mice pretreated with antibiotics. FMT from donors that consumed Goji berries also protected against elevations in markers of acute alcohol-induced liver injury in recipient mice.^34^ *Thymus quinquecostatus* Celak extract (TQE) is a species of thyme, widely used as food additive in Asia, that possesses hepatoprotective activity.^37^ To investigate the mechanisms of TQE’s liver protective effects in vivo, TQE supplementation alleviated chronic alcohol-induced liver injury and markers of gut barrier dysfunction in mice, likely through suppression of toll-like receptor 4-mediated inflammatory response and overproduction of reactive oxygen species.^32^ FMT studies using material from TQE-exposed donors also counteracted the alcohol-induced gut dysbiosis and partially ameliorated liver injury in the recipient mice, suggesting a causal role of the gut-liver axis in the hepatoprotective effects of TQE.^32^ Together, these studies show hepatoprotective effects of dietary supplements on acute or chronic alcohol-induced liver disease. FMT was used to show that these hepatoprotective effects can be transferrable and show causal role of the gut-liver axis in models of ALD.

### Gut Microbiome and Alcohol Consumption: Preclinical Studies

Few studies have used preclinical models to directly investigate the role of the gut microbiome on alcohol drinking or alcohol-related phenotypes such as anxiety and depression.^38^–^43^ Some of these studies used cross-species FMT to establish causality of the gut microbiome on alcohol drinking and related behavior.^38^,^40^–^42^ Most of these six studies investigated the effect of microbiomes after alcohol exposure on similar outcomes and on gene or protein expression within the brain.^38^,^39^,^41^–^43^ In one of the first studies directly assessing the ability of the gut microbiome to contribute to the development of alcohol-related behaviors, transplantation of gut microbiota from alcohol-fed mice facilitated the development of depressive-like behavior in alcohol-naïve recipients.^39^ In this model of noncontingent voluntary alcohol consumption, 4 weeks of escalating ethanol concentrations in the drinking water did not alter bacterial abundance but did change gut microbiota composition. Alcohol-exposed mice displayed signs of negative affective behavior following alcohol withdrawal in two rodent models of depression (i.e., the forced swim and tail suspension tasks). Additionally, they exhibited decreased expression of the brain-derived neurotrophic factor (*Bdnf*) and corticotropin-releasing hormone receptor 1 (*Crhr1*) genes, as well as increased expression of the mu opioid receptor (*Oprm1*) gene in the hippocampus. Fourteen days of daily FMT from alcohol-drinking mice into alcohol-naïve recipients (Alc-FMT) increased their depression-like behavior, similar to that of the alcohol-drinking donors. These findings were interpreted as transference of behavioral signs of alcohol withdrawal-induced negative affect. Additionally, similar gene expression changes in *Bdnf*, *Crhr1*, and *Oprm1* found in alcohol-exposed mice were seen in the hippocampus of Alc-FMT mice. Finally, as seen in previous studies, both alcohol consumption and alcohol-FMT decreased the relative abundance of *Lactobacillus* and increased *Allobaculum* abundance.^39^

To investigate whether changes in the gut microbiome are a cause or a consequence of alcohol drinking, Segovia-Rodriguez et al. treated alcohol-naïve rats with FMT from rats exposed to high (10 g/kg) ethanol doses (Alc-FMT), control-FMT, or phosphate-buffered saline control for 10 days.^40^ Antibiotic pretreatment was also tested in each group given the known effects of antibiotics on gut microbiome diversity and alcohol intake. Alc-FMT rats without antibiotic pretreatment increased their alcohol intake as compared to rats given control buffer via oral gavage, while control-FMT mice had decreased alcohol intake in the drinking in the dark multiple scheduled access model. The increased intake in Alc-FMT rats occurred 2 weeks after the last fecal transplant. The researchers suggested that this could be due to an interaction between the new Alc-FMT microbiota received and alcohol consumption, producing a synergistic effect that favored bacteria most benefited by alcohol consumption. Antibiotic pretreatment caused a significant reduction in alcohol consumption, and neither Alc-FMT nor control-FMT had an effect on intake. Additionally, spontaneous locomotor activity was reduced in the Alc-FMT mice, and antibiotic pretreatment abolished this effect.^40^ The findings suggest that, similar to the study by Ferrere et al.,^23^ alcohol preference may be dependent on the content of the gut microbiome since antibiotic pretreatment abolished the effects of both control-FMT and Alc-FMT.^40^

In another study not involving FMT, a dietary probiotic (*Lactobacillus rhamnosus* Gorbach-Goldin [LGG]) was used to modify the gut microbiota and assess alcohol intake in a rat model of alcohol relapse drinking.^42^ Rats selectively bred for alcohol drinking consumed alcohol for 5 weeks before they were administered antibiotics followed by daily LGG during a forced deprivation period. Antibiotic treatment alone led to a reduction (30%–40%) of early alcohol relapse drinking (i.e., within 60 minutes of restored access to alcohol), which increased to a 20% decrease of relapse drinking with 24-hour access. LGG treatment inhibited relapse drinking by 66% to 80%, as did administration of *N*-acetylcysteine + acetylsalicylic acid (NAC+ASA), which inhibits the alcohol-induced hyperglutamatergic condition. However, the combination of LGG and NAC+ASA during the deprivation period showed additive effects and virtually suppressed (90% inhibition) binge-like drinking after renewed access to alcohol. The reductions in alcohol deprivation effect were accompanied by differential alterations in protein levels in the nucleus accumbens. LGG treatment increased dopamine transporters, while NAC+ASA increased glutamate transporter levels (xCT and GLT-1), suggesting these dietary supplements are acting through different mechanisms to reduce alcohol relapse.^42^

### Role of Gut Microbiome in ALD: Clinical Studies

The gut microbiome—including bacteria, fungi, and viruses—has been implicated in the progression of liver disease in patients with underlying AUD; however, the few clinical studies that exist offer variable results.

#### Bacteria

A study by Maccioni et al. compared patients with ALD to healthy controls in an analysis of microbiota from feces and duodenal mucosa.^44^ In this study, patients with hepatic inflammation and fibrosis had increases in potentially pathogenic bacterial taxa, including *Streptococcus, Shuttleworthia*, and *Rothia*. This supports the notion that alcohol exposure increases intestinal permeability and that this can potentially contribute to ALD development, though further studies are warranted. Patients with alcohol-associated cirrhosis exhibit an increase in oral microbial species (*Lactobacillus salivarius, Veillonella parvula, Streptococcus salivarius*, and *Bifidobacterium*) in stool compared to controls and patients with alcohol use disorder without cirrhosis.^45^ Furthermore, pro-inflammatory bacteria such as *Enterobacteriaceae* were increased in patients with alcohol dependence, whereas butyrate-producing species (*Clostridiales*) were decreased.^45^ Specifically, cirrhosis was significantly associated with the presence of *Bifidobacterium*. The *B. dentium* species, linked to alcohol-associated cirrhosis, has been shown to play an important role in GABA production.^4^

Another study analyzed microbiota in the colons of healthy controls as well as 48 patients with AUD with and without liver disease.^46^ Mutlu et al. suggested that dysbiosis was worse in patients with alcohol-associated cirrhosis than in those with cirrhosis from other causes. Their study demonstrated that even in the early stages of ALD (without cirrhosis), changes in the gut microbiome occurred, such as reduced *Bacteroidetes* and increased *Proteobacteria*, and that levels of endotoxin were higher in patients who consumed alcohol.^46^ Alcohol also has been shown to decrease commensal taxa in patients consuming alcohol, irrespective of their cirrhosis status.^47^ It is suspected that increases in oral microbiota in the stool of patients with cirrhosis could be a result of the higher rate of oral infections, changes in salivary microbiome, and use of acid-lowering medications in this population. One study also suggested that increasing severity of liver disease is associated with a relative decrease in *Akkermansia muciniphila*.^48^ Therefore, changes to the gut microbiome may be influenced by the severity of liver disease.

#### Fungi

Studies in people with ALD have identified an increase in *Candida* species and a decrease in *Epicoccum, Galactomyces*, and *Debaryomyces*. Lower fungal diversity was observed in patients with ALD compared to healthy controls. In addition, these changes to the intestinal mycobiota were consistent among patients with varying degrees of ALD.^49^,^50^

#### Viruses

The link between viruses and ALD is complex, and current knowledge is limited.^51^,^52^ In patients diagnosed with alcohol-associated hepatitis, phages with hosts as varied as *Escherichia, Enterobacteria*, and *Enterococcus* were increased, as were viruses such as Parvoviridae and Herpesviridae. Specifically, the severity of ALD was associated with the presence of *Staphylococcus* phages and Herpesviridae.^52^

#### Effects of gut microbiota modulation

Several studies have assessed the effects of modulation of the gut microbiota on ALD. In a double-blind, placebo-controlled study, Amadieu et al. assigned a prebiotic (inulin) versus placebo for 17 days to 50 patients with ALD.^53^ Patients receiving inulin had significantly higher markers of hepatic inflammation. In the subset of patients who had early ALD (as defined based on FibroScan and serum values), inulin administration was linked to an increase in *Bifidobacterium* and a decrease in *Bacteroides*, and again, higher levels of hepatic inflammation. These findings suggest that inulin may be able to alter the gut microbiome but not necessarily lead to clinically apparent changes to inflammation and that prebiotics may not be successful or beneficial for improvement in liver parameters. This study was limited, however, by sample size and a relatively short duration of inulin administration. Another study assessed the effects of LGG use in patients with moderately severe alcohol-associated hepatitis. LGG was associated with reduced short-term liver injury and reduction of alcohol consumption to abstinence levels at 6 months.^54^

The role of SCFAs also has been explored in patients with ALD. A metabolomics analysis of fecal specimens demonstrated changes in tetradecane, reduced antioxidant fatty alcohols, and reduced SCFAs.^55^ These alterations promote an environment prone to oxidative stress and increased gut permeability.

### Role of FMT in AUD Treatment

Another area of interest has been the role of FMT in AUD treatment. Bajaj et al. demonstrated the safety of FMT in patients with alcohol-associated cirrhosis.^56^ They concluded that FMT was associated with reduced alcohol consumption and craving, with higher SCFA and microbial diversity. There was also a nonsignificant trend toward abstinence in the FMT group. Wolstenholme et al. further explored these mechanisms in a cross-species FMT design, mentioned below.^41^ A larger trial studying the clinical efficacy of FMT (NCT05548452) is currently enrolling.^57^

To extend these findings, Philips et al. treated patients with severe alcohol-associated hepatitis with FMT and prospectively analyzed stool samples.^58^ During a follow-up period of up to 3 years, patients who underwent FMT had lower rates of ascites, encephalopathy, infections, and hospitalizations with higher survival rates. Moreover, the FMT group demonstrated decreased alcohol relapse rates and longer time to relapse when compared to the standard-of-care group. Regarding microbiota composition, the FMT group demonstrated an increase in *Bifidobacterium* and a decrease in *Acinetobacter*, thus favoring a nonpathogenic milieu.

In patients with severe alcohol-associated hepatitis refractory to steroid therapy, liver transplantation, with the limitations described above, typically is the next treatment option. To address this, an open-label study was conducted with eight patients who were ineligible for steroid therapy and were treated with nasojejunal FMT for 1 week.^59^ Patients treated with FMT were found to have higher transplant-free survival, associated with reduction in pathogenic bacteria, as compared to historical patients with steroid-refractory alcohol-associated hepatitis (87% vs. 33%). Specifically, at the 1-year follow-up, patients treated with FMT had fewer *Proteobacteria* and more *Actinobacteria*. Furthermore, they exhibited a relative increase in nonpathogenic bacteria such as *Enterococcus villorum* and *Bifidobacterium longum*. Notably, there was coexistence of recipient and donor species at 6 and 12 months after FMT.^59^

The benefit of steroid treatments for severe alcohol-associated hepatitis is modest and limited to 28-day survival. Patients with alcohol-associated hepatitis have microbiota changes characterized by predominance of pathogenic species leading to immune dysregulation. Another study comparing FMT in 13 patients with standard of care (without steroids) in 20 patients reported a statistically significant increase in 90-day survival with FMT (54% vs. 25%, *p* = 0.02).^60^ In an extension of these two studies,^59^,^60^ Pande et al. compared the safety and efficacy of healthy-donor FMT versus prednisolone therapy in patients with severe alcohol-associated hepatitis in an open-label study; each group included 60 patients.^61^ There was a statistically significant improvement in 90-day survival in the FMT group compared to the prednisolone group (75% vs. 57%, p = .044). Moreover, there were significantly fewer deaths related to infections in the FMT group, suggesting that FMT can be a safe alternative in patients with severe alcohol-associated hepatitis. However, further studies are needed with differing formulations.

### FMT and Gut-Brain Axis Changes in ALD: Clinical Studies

A randomized controlled trial of FMT enema of men with cirrhosis and recurrent hepatic encephalopathy found that FMT increased microbiota diversity and improved cognition compared with standard of care.^62^ Using a rationally derived stool donor that was enriched in SCFA-producing *Lachnospiraceae* and *Ruminococcaceae*, this open-label randomized controlled trial with a follow-up period of 5 months found that with antibiotic pretreatment and administration of an FMT enema, the FMT was significantly better tolerated than the standard of care treatment^62^ Whereas five patients in the standard of care group developed hepatic encephalopathy, none of the patients who had received FMT did. Other benefits associated with FMT included improved cognitive performance and changes in the microbiome, such as relative reduction in nonpathogenic taxa and increased microbial diversity.^62^ A subanalysis of the data showed that improvement in microbial function was linked to cognitive improvement.^63^ Long-term follow-up of participants in this trial showed a continued relative increase in *Burkholderiaceae* and decrease in *Acidaminococcaceae* in the FMT group.^64^ Furthermore, the FMT group had decreased rates of liver-related hospitalizations and hepatic encephalopathy recurrence, suggesting that FMT could significantly improve the clinical course of patients with cirrhosis and have a positive impact on quality of life as well as reduce the economic burden of hospitalization.^64^

The effect of orally administered FMT on the gut-brain axis in cirrhosis also was studied in a phase I, randomized, placebo-controlled trial. Cognitive function improved after FMT, as measured by performance using the EncephalApp.^65^ The study also confirmed the primary endpoint of safety and tolerability of the oral FMT capsules.^65^ FMT also improved mucosal diversity, dysbiosis, and microbial function.^66^

#### Cross-species studies of microbiota and AUD

In one of the first cross-species studies, the gut microbiota from patients with AUD increased alcohol preference, induced changes in anxiety-like and depression-like behaviors, and altered brain gene expression of recipient mice.^38^ The fecal microbiome of men hospitalized for AUD (Alc-FMT), enriched in *Firmicutes* and *Bacteroidetes*, or of the control group of men who had abstained from alcohol for at least a year (control-FMT) was transplanted over 13 days into male mice that had been pretreated with antibiotics. Alcohol intake and preference for 4% or 8% alcohol in a two-bottle choice model were increased in the Alc-FMT mice compared to control-FMT mice. Alc-FMT mice also showed decreased anxiety-like behavior (indicated by increased time in the open arms of the elevated plus maze or in the center of an open field), increased depression-like behavior (indicated by immobility in the tail suspension test), and fewer social interactions compared to control-FMT mice. With respect to gene expression, Alc-FMT mice showed reduced expression of the metabotropic glutamate receptor 1 (*mGluR1*) and *PKCɛ* mRNA in the nucleus accumbens and reduced *Bdnf* and GABA_A_ receptor (alpha-1GABA_A_R) expression in the medial prefrontal cortex. Of note, antibiotic treatment prior to FMT modified some behaviors (e.g., decreased anxiety-like behavior) and increased locomotor activity in some tasks; however, social interactions and depressive-like behavior were not altered. Overall, the findings demonstrated that the gut microbiome of heavy drinkers can transmit some behavioral phenotypes similar to those seen in human drinkers.^38^

A separate study extended these cross-species findings by investigating the effects of an alcohol-FMT on addiction-associated behaviors such as sociability, anxiety-like and depression-like behavior; on brain functions such as myelination, neurotransmission, and inflammation; and on intestinal bacterial load and permeability.^43^ Mice that received an FMT from patients with AUD with severe symptoms of gut dysbiosis; high depression, anxiety, and alcohol craving; and low sociability also displayed deficits in a social preference task and higher depressive-like behavior; however, no differences were found in models of anxiety-like behavior.^43^ This was accompanied by increased corticosterone levels compared to mice that received control FMT. Within the brains of Alc-FMT mice, expression of several neurotransmitter subunits and myelin-associated genes was altered, but pro-inflammatory cytokines, chemokines, and markers of microglial activation were increased in the striatum, but not the prefrontal cortex, suggesting a local inflammatory response. Total bacterial load in the intestine was reduced in Alc-FMT mice, suggesting a lower bacterial count. The relative abundance of *Bacteroidetes* was decreased, while the abundance of *Firmicutes* was increased, similar to what is found in patients with AUD. This was accompanied by indicators of increased intestinal permeability, including decreased expression of markers of defense immune mechanisms, loss of intestinal homeostasis (reduced expression of *Reg3g* and *Lcn2*), modification of tight junction expression, and atrophy of the mucosal structure (reduced villous height and crypt depth in the ileum). Interestingly, the study suggested that the behavioral changes may not have been induced through a peripheral inflammatory response, but rather may have been a result of blood metabolite changes. Although the FMT-treated mice were not exposed to alcohol, increased portal vein ethanol concentrations were found in Alc-FMT mice. This suggests that the Alc-FMT mice likely were colonized by higher amounts of alcohol-producing bacteria such as *Clostridium, Lactococcus, Turicibacter*, and *Akkermansia*.

In a third study using a cross-species FMT design, changes in alcohol preference and intake that occurred in patients with AUD after receiving a fecal transplant were transmissible by FMT to germ-free mice (i.e., which had been treated to lack any microorganisms).^41^ The study used fecal samples from a randomized clinical trial that demonstrated reduced alcohol craving and consumption after fecal transplantation in patients with severe AUD. Germ-free male mice then received either stool or sterile supernatants (the nonmicrobial buffer collected from around the stool pellet) collected from trial participants pre-/post-fecal transplant. Mice colonized with postfecal transplant stool but not supernatants exhibited reduced alcohol acceptance, intake, and preference compared with mice receiving prefecal transplant stool. Analyses of gene expression in the liver, intestine, and prefrontal cortex revealed that a majority of the differentially expressed genes—which were related to immune response, inflammation, oxidative stress response, and epithelial cell proliferation—occurred in the intestine rather than in the liver or prefrontal cortex.^41^ These findings suggest a potential for therapeutically targeting gut microbiota and the microbial-intestinal interface to alter gut-liver-brain axis and reduce alcohol consumption in humans.

## Conclusions and Future Directions

The studies reviewed here demonstrate the role of the gut microbiome in AUD and ALD. They suggest that the use of probiotics, prebiotics, or FMT warrants further investigation as therapeutic approaches for these conditions. In clinical and preclinical studies, excessive drinking or exposure to high levels of alcohol was associated with dysbiosis, intestinal permeability, and changes in immune response (see Table 1). Clinical studies have suggested that use of FMT in patients with AUD improved SCFA levels, which may reduce inflammation and aid in preventing additional liver damage.^56^ FMT also has recently been used in preclinical models to manipulate the gut liver axis with certain dietary supplements to alleviate signs of acute or chronic alcohol-induced liver disease.^32^–^34^

Preclinical studies have used probiotics, prebiotics, or FMT from animals that had consumed those substances to improve alcohol-related behaviors such as alcohol consumption, providing evidence that gut microbiome manipulation may improve not only inflammation-related markers, but alcohol-related behaviors as well.^41^,^42^ Several of the preclinical studies identified in this narrative review were proof-of-concept FMT studies to show that behaviors such as anxiety-like and depression-like phenotypes and alcohol drinking can be induced by FMT from a donor with a history of alcohol exposure.^38^–^40^,^67^ However, the body of evidence in regards to FMT studies currently is still limited.

Clinical data suggest that with strict donor screening protocols, FMT appears to be safe, with low incidence of reported adverse events; however, long-term prospective data are still lacking.^56^ Currently, FMT only is indicated for recurrent *Clostridium difficile* infection, but the mounting evidence from preclinical and clinical studies suggests that it may be a therapeutic option for ALD as well.^47^ Several challenges exist, however, including the need to define a healthy stool donor, determine the optimal route of FMT administration, and find effective ways to validate endpoints. Changes in the microbiome can lead to progression of ALD by maintaining a state of localized and systemic inflammation.^46^ Also, although human studies support the role of healthy-donor FMT in improving transplant-free survival, reducing rates of infections, and even ameliorating craving for alcohol in patients with AUD, clinical data are limited by small sample sizes. Moreover, these studies often have focused on advanced ALD, and the benefit of FMT intervention on the liver and on psychological parameters in patients with less advanced forms of ALD remains unknown.

A study that compared pentoxifylline, corticosteroid, and nutritional therapy with FMT found that patients who received FMT via nasojejunal route had the highest survival rates of all groups at 3-month follow-up, suggesting a possible mortality benefit for FMT. FMT also led to improvement in clinical parameters while modulating and targeting inflammatory pathways such as LPS.^68^ Therefore, when compared to other medical interventions such as steroids that have side effects, FMT could potentially serve as a relatively benign treatment modality. However, a major limitation to this study was inclusion of only male patients, which raises the question of generalizability.

Understanding of the role of the microbiome in progression of ALD is growing rapidly. However, questions remain regarding its exact role in the pathophysiology of liver disease and in therapeutic strategies. Although abstinence remains the cornerstone of therapy for AUD, the point at which abstinence can modulate changes at the microbiome level is poorly understood. Future studies should focus on the composition and function of the microbiome and its byproducts at the various stages of the ALD spectrum. This will require large, prospective clinical trials with a diverse population sample. Although preclinical studies have suggested that manipulation of the gut microbiome may alter drinking behavior, few clinical trials of microbiome-targeted interventions have assessed drinking behavior as an endpoint. Such studies would be important in assessing the impacts of FMT on AUD outcomes outside of ALD. The gut-brain axis also is known to play a critical role in AUD, as demonstrated by individuals with AUD having increased gut permeability that leads to higher rates of depression, anxiety, and alcohol craving after a short period of abstinence.^12^ These observations suggest the microbiota can modulate cravings and other psychiatric comorbidities associated with addictive behaviors.

Dysbiosis occurs in some patients across the spectrum of liver disease severity, and changes in the microbiome are evident at the bacterial, viral, and fungal community level. Probiotics may address these changes; however, although probiotics have been associated with improvements in direct and indirect markers of disease severity in patients with ALD, most studies only had a small sample size, had a heterogeneous trial design, and were rarely reproduced. Targeting bacterial metabolites also could be promising, and given that patients with ALD have reduced levels of total fecal bile acids and SCFAs, addressing these changes could be a potential therapeutic target.

In summary, this review highlights the fact that, to date, few studies have evaluated FMT as a therapeutic option for reducing symptoms associated with excessive alcohol use. However, the number of such investigations is growing, and early studies have shown remarkable potential with a good safety profile. Although additional, larger clinical studies still are needed to determine whether FMT is an effective therapeutic strategy, evidence to date suggests that targeting the gut microbiome could be a promising treatment option for decreasing the risk of relapse in AUD patients and ameliorating the severity of ALD.

## Figures and Tables

**Table 1:** Summary of Preclinical and Clinical Studies Assessing the Effects of Fecal Microbiota Transplant (FMT) on Alcohol-Related Outcomes

Study*	Subjects	Model	Main Finding
Ferrere et al. (2017)^23^	Mice	Signs of ALD lesions after Lieber-DeCarli diet	FMT prevented the development of alcohol-induced liver lesions, but the effect depended on the host microbiome.
Wrzosek et al. (2021)^30^	Mice	Signs of ALD after FMT from SAH patients	Pectin-FMT beneficially reshaped the GM, in an AhR-dependent manner.
Yu et al. (2020)^31^	Mice	Signs of ALD lesions after Lieber-DeCarli diet with ethanol	FMT or LRP6-CRISPR improved GM diversity and composition to ameliorate ALD symptoms.
Yan et al. (2021)^32^	Mice	Signs of ALD lesions after Lieber-DeCarli diet with ethanol	TQE supplementation or TQE-FMT alleviated chronic alcohol-induced liver injury and markers of gut barrier dysfunction.
Yan et al. (2021)^33^	Mice	Signs of ALD lesions after Lieber-DeCarli diet with ethanol	UA had hepatoprotective effects and suppressed alcohol-induced oxidative stress and intestinal barrier disruption.
Guo et al. (2022)^34^	Mice	Acute ALD signs by ethanol lavage	Goji berries restored intestinal epithelial cell integrity and prevented acute liver injury induced by alcohol intake.
Xiao et al. (2018)^39^	Mice	FMT from noncontingent drinking mice	Alc-FMT transferred negative affective behaviors following withdrawal, altered brain gene expression, and reduced GM diversity.
Segovia-Rodriguez et al. (2022)^40^	Rats	FMT from ethanol-exposed rats (10 g/kg for 10 days)	Alc-FMT increased drinking and reduced locomotor activity, but this was dependent on antibiotics pretreatment.
Ezquer et al. (2022)^42^	Alcohol-preferring rats	Alcohol relapse drinking and LGG treatment	LGG modified the GM, reduced alcohol intake, and altered brain protein expression in a model of relapse drinking.
Bajaj et al. (2021)^56^	Humans	Patients with alcohol-associated cirrhosis and AUD	FMT reduced alcohol consumption and cravings and increased microbial diversity.
Philips et al. (2022)^58^	Humans	SAH hepatitis patients	FMT decreased alcohol relapse rates and increased time to relapse, increased beneficial GM diversity, and lowered rates of infections and hospitalizations with higher survival rates.
Philips et al. (2017)^59^	Humans	Open-label study of patients ineligible for steroid therapy	FMT recipients had higher transplant-free survival associated with reduction in pathogenic bacteria.
Sharma et al. (2022)^60^	Humans	Open-lab nonrandomized trial with severe alcohol-associated hepatitis with ACLF	FMT significantly reduced 28- and 90-day mortality and inflammatory cytokines.
Bajaj et al. (2017)^62^	Humans	Open-label randomized trial: outpatient men with cirrhosis and recurrent HE received FMT enema	Improved cognition along with increased microbial diversity.
Bajaj et al. (2019)^65^	Humans	Randomized, single-blind study: cirrhosis with recurrent HE receiving FMT capsules vs. placebo	FMT capsules were safe and improved duodenal mucosal diversity, dysbiosis, and objective measures of encephalopathy.
Philips et al. (2018)^68^	Humans	Comparative study between pentoxifylline, corticosteroid, nutritional therapy, and FMT	FMT had highest survival rates at 3-month follow-up by modulating GM composition and function and decreasing inflammatory pathways.
Zhao et al. (2020)^38^	Humans to mice	Cross-species Alc-FMT	Human to mouse Alc-FMT increased alcohol preference and negative affective behaviors and altered brain gene expression.
Wolstenholme et al. (2022)^41^	Humans to mice	Cross-species Alc-FMT and treated Alc-FMT	Alcohol preference and intake were reduced in patients with AUD after receiving FMT, and this behavior was transmissible to mice; liver, intestine, and brain gene expression was altered in mice.
Leclercq et al. (2020)^43^	Humans to mice	Cross-species Alc-FMT	Human-to-mouse Alc-FMT increased depression-like behavior and lowered sociability; brain neurotransmitter and myelin gene expression were altered.

Studies are ordered by citation number within each subject type.

*Note:* ACLF, acute-on-chronic liver failure; AhR, aryl hydrocarbon receptor; Alc, alcohol; ALD, alcohol-associated liver disease; AUD, alcohol use disorder; CRISPR, clustered regularly interspaced short palindromic repeats; FMT, fecal microbiota transplant; GM, gut microbiota; HE, hepatic encephalopathy; LGG, *Lactobacillus rhamnosus* Gorbach-Goldin; LRP6, low-density lipoprotein-related protein 6; SAH, severe alcohol-associated hepatitis; TQE, *Thymus quinquecostatus* Celak extract; UA, ursolic acid.
